# Elimination of onchocerciasis from Colombia: first proof of concept of river blindness elimination in the world

**DOI:** 10.1186/s13071-018-2821-9

**Published:** 2018-04-11

**Authors:** Rubén Santiago Nicholls, Sofía Duque, Luz Adriana Olaya, Myriam Consuelo López, Sol Beatriz Sánchez, Alba Lucía Morales, Gloria Inés Palma

**Affiliations:** 1 Grupo de Parasitología, Instituto Nacional de Salud, Avenida Calle 26 No. 51–20, Bogotá, DC CP 111321 Colombia; 2 Laboratorio Departamental de Salud Pública, Secretaría Departamental de Salud del Cauca, Calle 5 No. 15–57, Popayán, Cauca CP 190003 Colombia; 3 Departamento de Salud Pública, Facultad de Medicina, Universidad Nacional de Colombia, Ciudad Universitaria, Bogotá, DC CP 111321 Colombia; 4 Consultant Health Education, Colombian Onchocerciasis Elimination Program, Carrera 93 # 16–90, Cali, CP 760032 Colombia; 5 Onchocerciasis Elimination Program for the Americas (OEPA), 14 calle 3–51, zona 10, Edificio Murano Center, CP 01010 Guatemala, Guatemala; 6 Departamento de Microbiologia, Facultad de Salud, Universidad del Valle, Campus San Fernando, Calle 4B No. 36–00, CP 760043 Cali, Colombia

**Keywords:** Onchocerciasis, Colombia, Elimination, Infectivity rate, Blackfly, Ivermectin

## Abstract

**Background:**

Onchocerciasis is a chronic parasitic infection originally endemic in 13 discrete regional foci distributed among six countries of Latin America (Brazil, Colombia, Ecuador, Guatemala, Mexico and Venezuela). In Colombia, this disease was discovered in 1965 in the Pacific Coast of the country. The National Onchocerciasis Elimination Program was established in 1993 with the aim of eliminating disease morbidity and infection transmission. In 2013, the World Health Organization (WHO) verified Colombia as free of onchocerciasis, becoming the first country in the world to reach such a goal. This report provides the empirical evidence of the elimination of *Onchocerca volvulus* transmission by *Simulium exiguum* (*s.l.*) after 12 years of 6-monthly mass drug administration of Mectizan® (ivermectin) to all the eligible residents living in this endemic area.

**Methods:**

From 1996 onwards, a biannual community-based mass ivermectin administration programme was implemented, complemented by health education and community participation. In-depth parasitological, serological and entomological surveys were conducted periodically between 1998 and 2007 to evaluate the impact of ivermectin treatment according to the 2001 WHO guidelines. When the interruption of parasite transmission was demonstrated, the drug distribution ceased and a three-year post-treatment surveillance (PTS) period (2008–2010) was initiated.

**Results:**

After 23 rounds of treatment, parasitological and ophthalmological assessments showed absence of microfilariae in skin and anterior chamber of the eyes. Serological tests proved lack of antibodies against *O. volvulus* in children under 10 years-old. A total of 10,500 *S. exiguum* flies tested by PCR had no L3 infection (infectivity rate = 0.0095%; 95% CI: 0.0029–0.049) during 2004, indicating interruption of parasite transmission. However, biannual ivermectin treatments continued until 2007 followed by a 3-year PTS period at the end of which 13,481 flies were analyzed and no infective flies were found (infectivity rate = 0%; 95% CI: 0.0–0.014).

**Conclusions:**

These results fulfilled the WHO criteria for onchocerciasis elimination. Consequently, in 2013 Colombia was verified as free of onchocerciasis, demonstrating that elimination of this neglected tropical disease is an achievable goal and paving the way for an elimination agenda to be followed by other endemic countries in Latin America and Africa.

## Background

Onchocerciasis is a chronic infection of humans caused by the filarial worm *Onchocerca volvulus* (Leuckart) and transmitted through the bites of infected females of blackfly species of the genus *Simulium* Latreille. The parasite’s embryonic forms, microfilariae (Mf), migrate through the skin and cause severe itching, disfiguring skin and ocular lesions, producing visual loss and blindness in patients with heavy parasite loads. In Latin America, 13 onchocerciasis foci were formerly prevalent in Brazil, Colombia, Ecuador, Guatemala, Mexico and Venezuela, where around 570,000 people were considered at risk of infection as of 2017 [1].

Based on the Pan American Health Organization’s Directing Council Resolution CD35R.14 [2], the Onchocerciasis Elimination Program for the Americas (OEPA) was established in 1992 with the primary purpose of acting as a technical and coordinating organization at the regional level to guide countries to achieve the goal of eliminating onchocerciasis in Latin America [3, 4]. This regional public health strategy specifically included elimination of new (ocular) morbidity caused by *O. volvulus* and interruption of transmission by 6-monthly mass administration of ivermectin (Mectizan®, Merck & Co. Inc.) with coverage (proportion of the population treated) equal to or higher than 85% of the eligible population [4]. Ivermectin is a drug that kills the Mf in the skin (microfilaricide) and temporarily inhibits their release by gravid adult worms [5, 6]. Hence, the elimination strategy has been based on safe and effective high treatment coverage for several years (due to the long life-cycle of the adult parasite) and for more than one cycle per year. The onchocerciasis elimination programme in Colombia, as those in the other five endemic Latin American countries, relied on this health strategy since 1996.

There is evidence that onchocerciasis was introduced in Colombia in the 17th and 18th centuries through the slave trade [7]. The first confirmed case of onchocerciasis in Colombia was described incidentally in 1965 [8]. The patient was born and had lived for most of his life in a village along the Micay River, near a town called Lopez de Micay (2°51'0"N, 77°15'2"W) located 120 km south from Buenaventura on the Pacific Coastal Plain (altitude, 50 m above sea level), at the foot of the West Andes (Cordillera Occidental) in Colombia [9]. This first case stimulated a series of epidemiological, parasitological, entomological, clinical and ophthalmological studies carried out between 1965 and 1970 in Lopez and its surrounding villages. These studies confirmed: (i) the presence of infection by *Onchocerca volvulus*, mainly in people coming from villages on the Micay River upstream from Lopez de Micay; (ii) the relatively low concentration of microfilariae in skin snips [9]; (iii) the mild nature of both skin disease and ocular alterations [9, 10]; and (iv) the role of the main predominant man-biting simuliid species, *Simulium exiguum* (*s.l.*), as the vector species [9–11]. Experimental studies showed that *S. exiguum* (*s.l.*) had limited vectorial efficiency, albeit sufficient to maintain transmission [12].

Two other epidemiological studies were carried out in 1977 and 1989; the results led to the conclusion that, although transmission persisted, the prevalence was apparently declining from 15.9% in 1965 [9] to 7.5% in 1977 [13] and 4.0% in 1989 [14] without any deliberate interventions specifically aimed at reducing the risk or at preventing transmission.

Following the creation of OEPA, the National Onchocerciasis Committee was established in 1993. In 1995, a baseline epidemiological assessment was carried out [15]. Mobile teams visited all the villages along the Micay River and its tributaries. Transmission was documented in a single village, Naicioná, where the prevalence in the population aged 15 years or older was 40% [15]. In this same year the possibility of a second transmission focus in the rural area of Tumaco, on the border with Ecuador, was ruled out [15]. This was further confirmed by the negative results of a rapid epidemiologic assessment carried out in Ecuador in 19 communities along or adjacent to the Mataje River which forms the border between Colombia and Ecuador [16]. Thus, it was concluded that onchocerciasis in Colombia was confined to the single focus of Lopez de Micay, specifically to the village of Naicioná, on the Pacific Coastal Plain of the country, along the Micay River basin. Here, about 1200 Afro-Colombian people were at risk, approximately 500 of them living in Naicioná and the remaining 700 natives of Naicioná living in downstreanm communities and in the city of Buenaventura. Parasite infection was transmitted by *Simulium exiguum* Roubaud (*s.l.*) [15].

Based on these results, an elimination programme was established in 1996. The main strategy was biannual ivermectin (Mectizan®) distribution to all the at-risk population, complemented by health education and the promotion of community participation.

The present work reports the elimination of *O. volvulus* transmission in the village of Naicioná, Lopez de Micay focus after 12 continuous years of ivermectin treatment. Baseline and further clinical, parasitological, ophthalmological and entomological evaluations carried out periodically in this community allowed to monitor the impact of ivermectin administration on the transmission of *O. volvulus* by *S. exiguum*.

## Methods

### Study area and study population

In Colombia, the onchocerciasis transmission comprised an area localized in the Chocó-Darién moist forests’ ecoregion, 60–100 m above sea level (masl) of the Micay River basin at the Pacific coastal zone of the country, the village of Naicioná (coordinates: 2°55'0"N, 76°55'00"W). This is a region of dense tropical rainforest and heavy annual rainfall (from 4000–8000 mm), with a maximum mean temperature of 30 °C, a minimum of 19 °C, and a very short dry season, generally from January to March. In this region, the at-risk population is the Afro-Colombian rural population dedicated to agricultural, hunting, fishing and mining activities [15].

### Ivermectin mass drug administration (MDA)

A periodic community-based ivermectin (Mectizan®) mass drug administration distribution (MDA) programme, complemented by health education and the promotion of community participation, began in September 1996 and continued uninterrupted until the end of 2007. Specifically, ivermectin distribution was initially limited to Naicioná but gradually expanded to include the nearby downstream communities of Playagrande and San Antonio de Chuare, gold panners along the Chuare River and the natives of Naicioná residing in the towns of Lopez de Micay and Buenaventura. Properly trained community health workers were responsible for the biannual distribution of treatments. Directly supervised single dose (according to weight or height) ivermectin treatments were administered twice a year from September 1996 to November 2007, to at least 85% of all the eligible population (children under five years of age, 90 cm height or 15 kg weight; pregnant women and severely ill people were considered ineligible). This was accompanied by health education, social mobilization and community participation for 14 consecutive years. Epidemiological coverage rates were calculated after each treatment round.

### Impact assessment of the MDA programme

In-depth epidemiological assessments were carried out periodically (Table 1) and their results compared to those of the baseline studies, following the then WHO recommended guidelines and methods [17]. Interruption of *O. volvulus* transmission is defined as the reduction of parasite infection to such levels (below specific parasite density breakpoints) that local transmission can no longer sustain the population [17]. Therefore, transmission is regarded as interrupted when the endemic focus has reached specific epidemiological indicators such as: (i) prevalence of < 1% of *O. volvulus* Mf in the skin and/or eye; (ii) a reduction of new infections to an incidence rate of less than one new case per 1000 individuals (< 0.1%) defined as lack of specific Ov-16 antibodies to *O. volvulus* in school children; (iii) an infectivity rate (L3 infection in heads) by PCR of < 1/1000 (0.1%) in parous flies or < 1/2000 (0.05%) in all flies, assuming a 50% parous rate; and (iv) an annual transmission potential (ATP) or a seasonal transmission potential (STP) under 20 L3s per time period.

**Table 1:** Calendar of the baseline and follow-up impact assessments carried out in Naicioná, Colombia

Component	Baseline assessment	First impact assessment	Second impact assessment	Third impact assessment	Fourth impact assessment
Parasitology	1995	1998	2001	2004	2007
Ophthalmology	1996	1998	2001	2006	No
Entomology	1996	1998	2001	2004	2010^a^
Serology	No	No	2001	2004	2007

^a^The entomological assessment in 2010 was done to confirm the interruption of transmission at the end of the 3-year post-treatment surveillance period

### Parasitological assessments

Skin snips were taken from the right scapula and right iliac crest and examined following the same procedure as in the baseline study [15]. The microfilariae prevalence in skin snips and the community microfilarial load (CMFL) was obtained.

### Ophthalmological assessments

Four ophthalmological assessments were done in order to establish the magnitude of ocular damage caused by *O. volvulus* and the impact of repeated ivermectin MDA treatments on ocular morbidity. The first three, in 1996 (baseline), 1998 and 2001, were carried out following the criteria and procedures recommended at that time by OEPA [18]. The last one in 2006 used the definition for onchocercal punctate keratitis described in the study by Winthrop et al. [19]. In the 1996 baseline study only persons with a microfilariae-positive skin snip were examined [15, 20], while in the other 3 studies all persons aged 10 years or older who were present at the time of the assessment were studied.

### Serological studies

Serology was carried out in children less than 10 years of age in 2001, 2004 and 2007. Blood samples were drawn by digital puncture with a sterile lancet. In 2001, capillary blood samples (collected by finger prick) were obtained from children and examined using Ov-16 card tests as described by Lipner et al. [21]. In 2004 and 2007 capillary blood samples were spotted onto filter paper and processed at the Centers for Disease Control (CDC) in Guatemala, using the ELISA technique for IgG4 antibodies against the Ov-16 recombinant antigen [22] following the methodology described by Linblade et al. [23] in 2007.

### Entomological studies

After the baseline studies, three entomological assessments were carried out to evaluate the impact of the MDA. All of them were completed during the second half of the year (July to December), the peak transmission season. Blackfly collections were done at known transmission sites in the community of Naicioná. Several sampling sites were selected. The first two entomological studies to assess the impact of MDA on transmission were carried out in 1998 and 2001, after four and ten treatment rounds, respectively. In 2001, some flies were dissected and others processed by polymerase chain reaction (PCR) to detect *O. volvulus* DNA [24]. For the 2004 survey, the 2001 WHO guidelines [17] for entomological studies to assess the impact of MDA on transmission were followed. Collections were carried out once a month for five consecutive months between July and November 2004. Of the collected blackflies, 10,500 were processed at OEPA’s Regional Reference Laboratory for polymerase chain reaction (PCR) (the laboratory of Dr Thomas Unnasch, currently at University of South Florida) to detect *O. volvulus* DNA as described by Katholi et al. [24]. The results were analyzed using the Poolscreen 2.0 program to determine the infectivity rate with a 95% confidence interval (95% CI) [24].

### Post-treatment surveillance (PTS)

When all the epidemiological indicators show that transmission has been interrupted, suspension of treatment is recommended and a 3-year period post-treatment surveillance is initiated in the focus [17]. Thus, the 3-year PTS was carried out between January 1st, 2008 and December 31st, 2010. Frequent contact, at least 4 times a year, was kept with the people living in Naicioná for health education, health promotion and prevention, nutrition, food security, social mobilization and annual updating of the census. Following the 2001 WHO guidelines [17], blackflies were collected in the second half of 2010, at the end of the PTS period, for several consecutive days once a month during 4 months in the peak transmission season (July to December) to confirm that transmission had not recrudesced in the absence of treatments. The female blackflies were processed at OEPA’s Regional Reference Laboratory for PCR as described above [24].

## Results

**Fig. 1:**
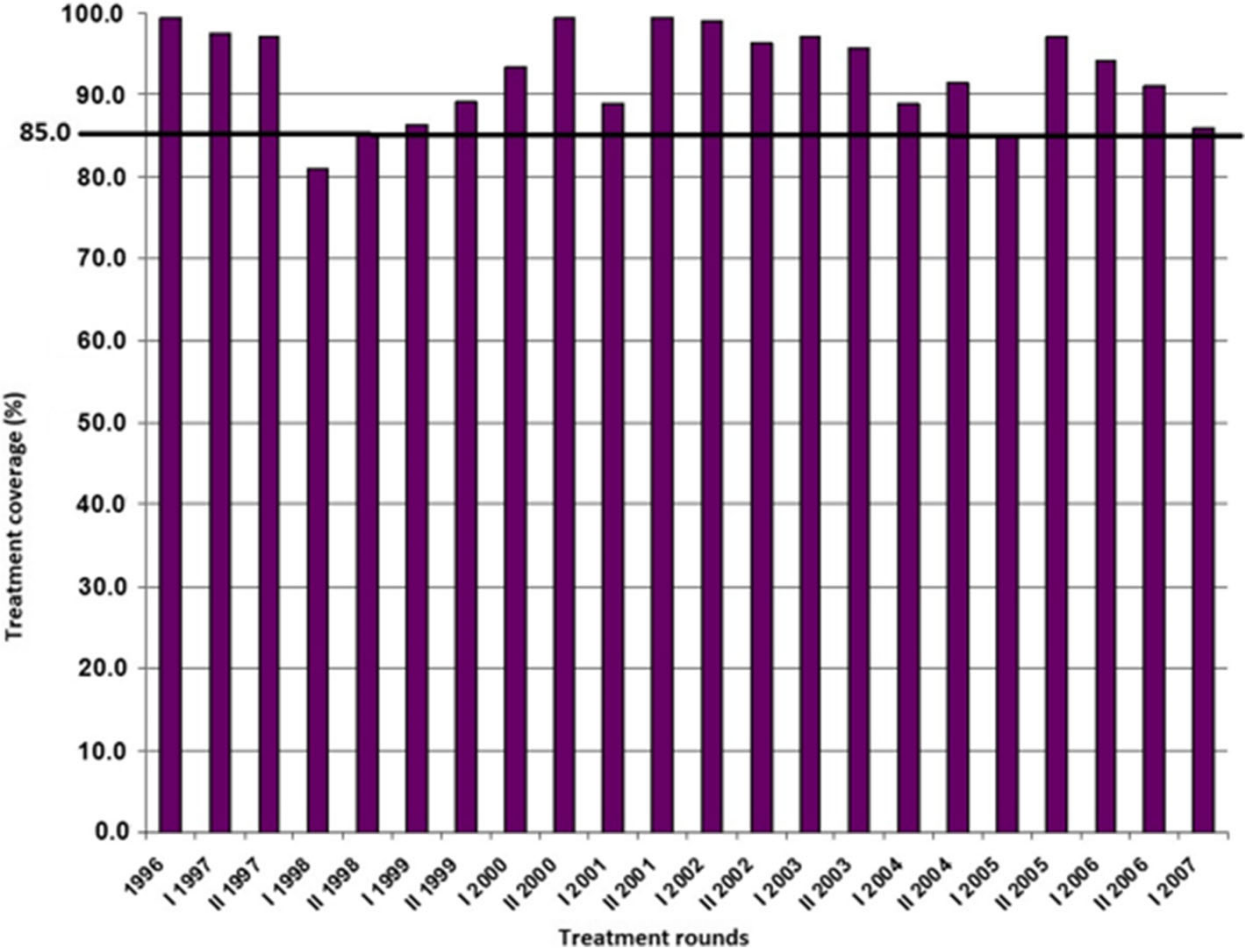
Historical (1996–2007) treatment coverage (%) of ivermectin (MDA) treatments, community of Naicioná, onchocerciasis focus, Colombia. The horizontal line at 85% indicates the minimum coverage of eligible people that needs to be reached and sustained to interrupt transmission according to OEPA’s strategy

The historical 6-monthly ivermectin treatment coverage (MDA period) in the community of Naicioná is presented in Fig. 1. Coverage of at least 85% of the eligible population was achieved in 1999 (for each treatment round) and was sustained since then. Up to the end of 2007, 23 consecutive treatment rounds were carried out.

Pre-treatment data in Naicioná showed a baseline 40% prevalence of microfilariae in skin snips in people aged 15 years or older, classified as a mesoendemic community according to OEPA guidelines. The initial infection intensity of the parasite infection in the community by contrast was relatively low and below 1, as measured by the community microfilarial load (Table 2). Parasite surveys conducted at various time points during the ivermectin MDA showed that the prevalence of *O. volvulus* Mf in the population aged 15 years or older declined markedly as compared to the 1995 baseline figure, becoming negative after 22 rounds of ivermectin (Table 2). Prevalence of *O. volvulus* Mf also declined in the population of all age groups from 6.6% in 1998 to negative in 2007 (Table 2).

**Table 2:** Prevalence of microfilariae in skin snips and community microfilarial load (CMfL), Naiciona, Colombia, 1995–2007. The 95% confidence intervals were calculated by the Poisson Exact method for proportions using the Stata 11® software

Year	No. of treatment rounds	Prevalence in skin snip biopsy (all age groups)			Prevalence in skin snip biopsy (15-year-old or older)			CMfL
		*n*/*N*	%	95% CI	*n*/*N*	%	95% CI	
1995^a^	0	na	na	na	36/91	40	27.7–54.8	0.64
1998	4	16/244	6.6	3.7–10.6	10/114	8.8	4.2–16.1	0.07
2001	10	0/143	0	0–2.6	0/91	0	0–4.0	0
2004	16	2/232	0.85	1.0–3.1	2/127	1.6	0.2–5.7	0.0039
2007	22	0/263	0	0–1.4	0/157	0	0–2.3	0

^a^Baseline data; only people aged 15 years or older were examined in 1995

*Abbreviations*: *na* not applicable, *95% CI* 95% confidence interval

Regarding the ocular morbidity indicators due to onchocerciasis (Table 3), no microfilariae in the anterior chamber (MfAC) were found in any of the persons analyzed in the community. However, there were considerable levels of prevalence of punctate keratitis that varied from 32.6% at the pre-treatment period to negative in 2007.

**Table 3:** Results of the ophthalmological assessments in Naiciona, Colombia, 1996–2006

Year	People examined	No. of people with punctate keratitis	Prevalence of punctate keratitis (%)	95% CI
1996 (baseline)	46	15	32.6	18.2–53.8
1998	147	47	32	23.5–42.5
2001	105	27	26	16.9–37.4
2006	187	0	0	0–1.9

*Abbreviations*: *95% CI* 95% confidence interval

All 21 samples from children aged up to 5 years and all 78 samples from children aged 5 to 14 years were negative in the Ov-16 seroprevalence surveys carried out during 2001. Similarly, all serology samples from children aged from 0 to 9 years, 79 in 2004 and 64 in 2007, were negative.

Table 4 presents data on infectivity rates of *S. exiguum* (*s.l.*) prior to MDA and after up to 12 rounds of treatment. During these three surveys, the baseline of *O. volvulus* infectivity rate in this vector species, as determined by manual dissection was above the transmission threshold of 1% (1.07%, 95% CI: 0.3–3.1%) but declined below this level after five rounds of treatment.

**Table 4:** Evolution of *Simulium exiguum* (*s.l.*) infectivity rate, determined by dissection in Naiciona, Colombia, 1996–2001

Year	Method	No. of flies examined	Flies with infective larvae (L3)	Infectivity rate (%)	95% CI
1996 (baseline)	Dissection	281	3	1.07	0.3–3.1
1998	Dissection	286	0	0	0–1.2
2001	Dissection	3371	1	0.03	7.5 × 10^-6^–0.2

*Abbreviations*: *95% CI* 95% confidence interval

Finally, three additional entomological surveys using PCR techniques to determine biting and infectivity rates of the vector species as well as its seasonal transmission potential were carried out between 2001 and 2010 (Table 5). The seasonal biting rates in Naicioná during 2001 were considerably high with values reaching up to almost 90,000 flies per person per transmission season. However, although more flies were collected during 2004 and 2010, the biting rate of this vector species dropped considerably by almost four times from the previously observed values (Table 5). Also, during 2001, although the infectivity rate of flies carrying *O. volvulus* was below the threshold of 1/2000 flies, the upper limit of the 95% confidence interval of the seasonal transmission potential was still above 20 L3/person/year, which is considered the breaking point for transmission. In 2010, three years after MDA ended, none of the pools of *S. exiguum* (*s.l.*) collected (13,481 flies) were found to be positive in the PCR assay. Therefore, both the infectivity rate and the STP were below the threshold of 1/2000 and 20 L3/person/season, respectively.

**Table 5:** Infectivity rate and transmission potential of *Simulium exiguum* (*s.l.*) by PCR in Naiciona, Colombia, 2001–2010

Year	No. of flies examined	Biting rate (95% CI)	Infectivity rate (%) (95% CI)	Transmission potential (95% CI)
2001	5,565	73,958 (67,897–80,543)	0.017 (0.0005–0.095)	12.6 (0.3–71.7)
2004	13,115	22,919 (21,068–24,921)	0.0095 (0.0029–0.049)	2.2 (0.07–11.2)
2010	13,481	20,983 (19,391–22,696)	0 (0–0.014)	0 (0–2.9)

*Abbreviation*: *95% CI* 95% confidence interval

## Discussion

The results show the success of the Onchocerciasis Elimination Program in Colombia based on periodic, sustained biannual treatments with ivermectin (Mectizan®), complemented by health education, social mobilization and community participation, as measured by entomological indicators of transmission and by clinical, parasitological and ophthalmological indicators of morbidity.

The entomological evidence shows that after 12 years of continuous ivermectin MDA the *O. volvulus* infection transmission levels declined below the specific parasite density breakpoint indicating that local transmission could no longer sustain the parasite population [17] and, consequently, *O. volvulus* transmission was eliminated in the Lopez de Micay onchocerciasis focus in Colombia. This provided the first proof of concept that elimination of onchocerciasis can be achieved by biannual MDA with ivermectin sustained for a period of 10 to 12 years.

A clear decrease in prevalence and CMfL occurred as a consequence of the periodic distribution of ivermectin. However, it must be noted that only persons of 15 years or older were examined in the baseline survey in 1995 (Rapid Epidemiological Assessment), while in the follow-up assessments children between 1 and 15 years, as well as adults, were examined. Although no people were found to be positive for microfilariae in 2001 after 10 treatment rounds, this could possibly be explained by the fact that a lower number of people were examined as compared to the previous follow-up assessments. In 2004, after 16 treatment rounds, only two adults were found to be positive for microfilariae. For different reasons, they had not received ivermectin during the previous three treatment rounds. In 2007 none of the 263 people examined had microfilariae positive skin snips.

Before the criteria for *O. volvulus* punctate keratitis were reviewed [19], prevalences between 26–33% were detected. In 2006, when the updated definition was applied, no cases of punctate keratitis attributable to *O. volvulus* were detected but non-onchocercal punctate keratitis lesions were observed in 27% of the people examined. These, as well as the punctate keratitis lesions encountered in the previous assessments, were most likely of a different, non-onchocercal etiology, caused by either infections or small traumatic lesions.

Based on the results of the last ophthalmological assessment in 2006, it was concluded that ocular morbidity attributable to *O. volvulus* infection was absent. Collectively, the seroprevalence results indicated that children were not exposed to infection by *O. volvulus* since at least 2001*.*

The results of the entomological assessment carried out in 2004 showed that transmission was successfully interrupted because the infectivity rate found in 2004 was below the threshold of 0.05% (1/2000) required for transmission to occur as established in the 2001 WHO guidelines and criteria [17]. Nevertheless, biannual ivermectin treatments continued mainly because, for administrative reasons, the results of the entomological assessment carried out in 2004 were not available until 2007.

The findings of all parasitological, ophthalmological, serological and entomological assessments, together with the historical information of the treatment coverage, were extensively reviewed and discussed by the Program Coordinating Committee (PCC) of OEPA in June 2007. The PCC concluded that transmission had been interrupted and recommended Colombia’s Ministry of Social Protection to stop the biannual MDA in this focus and begin the 3-year post-treatment surveillance (PTS) period, following WHO guidelines [17].

The results of the entomological assessment carried out in 2010, at the end of the PTS period, demonstrated that the WHO criteria [17] for elimination of onchocerciasis transmission in the Lopez de Micay focus were fulfilled and therefore that elimination of onchocerciasis in Colombia had been reached. Consequently, these results represented the key evidence to assure that no parasite-vector contact was occurring anymore in this community after three years following the end of ivermectin MDA.

This achievement was obtained through biannual MDA with ivermectin (Mectizan®) with high coverage rates in the twelve year period between 1996 and 2007. The remote location of Naicioná and the inherent difficulties for its geographical accessibility posed an important challenge to the programme. However, the dedicated work of community health workers who were trained for distributing Mectizan® to all the eligible population and the acceptance of the periodic treatments by the community made possible this relevant public health success. Maintaining the interest of the community throughout all those years was the result of a continued effort to carry out a health education, community participation and social mobilization programme which included onchocerciasis but went beyond to address other health concerns of the inhabitants of Naicioná such as hypertension, nutrition, soil-transmitted helminthiases and intestinal parasitoses, food security and alcoholism. Other issues related with their living conditions such as water and sanitation as well as literacy, education and conflict prevention and resolution were also addressed. The acceptance of the treatments with Mectizan® was also favored by its effect on intestinal helminths. People were very pleased to note that a few days after taking the ivermectin tablets they passed adult roundworms in their stools. As a result, after the first five treatment rounds people were looking forward to receiving treatment during those months of the year when the MDA was scheduled.

In 2016 the WHO updated the criteria and procedures for stopping mass drug administration and verifying elimination of human onchocerciasis [25]. However, the entomological criteria for interruption and elimination of transmission, i.e. an upper bound of the 95% confidence interval of the prevalence of L3 of less than 0.05% (< 1/2000) in all flies (assuming a parity rate of 50%) at the end of the 3- to 5-year post-treatment surveillance period, remained the same as in the 2001 guidelines [17]. Although the 2016 WHO guidelines [25] also included a serological criterion, i.e. overall prevalence of < 0.1% in children aged under 10 years of age, this requires a sample size of at least 2000 children. This was statistically impossible to meet in Colombia given the small size (1200) of the at-risk population. Nevertheless, serology was always negative in all the children tested.

## Conclusions

The success in eliminating onchocerciasis transmission provides proof of concept and ratifies the principle that biannual ivermectin treatments with coverage rates of at least 85% eventually blocks transmission because it suppresses microfilariae production. If sustained during several consecutive years covering the life-span of adult worms, the reservoir of infection will eventually die out, leading to the elimination of both disease and transmission [4–6]. This OEPA strategy has contributed to the success of onchocerciasis elimination in the Americas. Currently, no new cases of onchocerciasis associated to blindness have been reported in most of the OEPA region and ocular morbidity has been eliminated from eleven of the 13 previously endemic foci in the region. Parasite transmission has been interrupted and elimination has been reached in 11 of the 13 foci, where 538,517 people (94.63%) of the total population are no longer at risk of suffering this debilitating disease. WHO has verified the elimination of onchocerciasis in four of the six countries where the disease was formerly endemic. In 2013, Colombia was the first country in the world where elimination of onchocerciasis was verified by WHO [26], followed by Ecuador in 2014 [27] and Mexico in 2015 [28, 29]. Onchocerciasis was also eliminated from Guatemala in 2015 [30], verified by the WHO in 2016 [31]. Although the situation regarding onchocerciasis elimination in Africa presents a much greater challenge, recent promising reports show that mass administration of ivermectin on an annual or biannual basis is interrupting transmission in Uganda [32, 33], Northern Sudan [34], and Mali and Senegal [35], suggesting that the elimination goal is also feasible in Africa. Of the 13 original foci in the Americas, transmission is currently limited to the hard-to reach Yanomami Area (Amazonian focus), shared by Venezuela and Brazil, with an estimated population of approximately 30,000 people [1]. This is the greatest challenge for achieving elimination in the Americas Region. Nonetheless, recent evidence shows that transmission has been suppressed in 70% of the communities in the Venezuelan side of the Yanomami Area, or Venezuela’s southern focus [36]. Although the risk of onchocerciasis reemergence or reintroduction in Colombia due to immigration of infected individuals is considered very low, given the remote location of Naicioná, and also taking into account that onchocerciasis transmission was eliminated in the Esmeraldas focus in Ecuador, the nearest of the other 12 foci in the Americas, and migration of infected people from foci in Brazil and Venezuela seems very unlikely, surveillance to detect any possible reintroduction has been established and should be maintained until elimination of onchocerciasis is achieved throughout the entire Americas Region. A sustained 16-year effort was necessary to eliminate onchocerciasis from Colombia. This important achievement was possible with the valuable support of international partners such as OEPA, Merck’s Mectizan® Donation Program and the Pan American Health Organization (PAHO), among others, and the national leadership of Colombia’s Instituto Nacional de Salud with the support from the Colombian Ministry of Health and Social Protection, and the important interinstitutional and multidisciplinary collaboration with the Health Secretariat of the Cauca department, the National University of Colombia, and the Valle University. Thanks to all of them we can now say that Colombia is now, for the first time in nearly 300 years and hopefully forever, free from the risk of onchocerciasis.

## Abbreviations

95% CI: 95% confidence interval
ATP: Annual transmission potential
CDC: Centers for Diseases Control and Prevention
CMfL: Community microfilarial load
ELISA: Enzyme linked immunosorbent assay
masl: Meters above sea level
MDA: Mass drug administration
Mf: Microfilariae
MfAC: Microfilariae in the anterior chamber
OEPA: Onchocerciasis Elimination Program for the Americas
PAHO: Pan American Health Organization
PCC: Program Coordinating Committee (OEPA)
PCR: Polymerase chain reaction
PTS: Post-treatment surveillance
STP: Seasonal transmission potential
WHO: World Health Organization

## References

[1] World Health Organization (2017). Progress towards eliminating onchocerciasis in the WHO Region of the Americas: elimination of transmission in the north-east focus of the Bolivarian Republic of Venezuela. Wkly Epidemiol Rec..

[2] Pan American Health Organization. Multinational strategic plan of action toward onchocerciasis elimination in the Americas. 1991. Annex VI of PAHO Directing Council, XXXV meeting, Agenda Item 5.4. Report on the status of the eradication/elimination of certain diseases from the Region. Washington, DC, September 1991, CD35.R14. http://www1.paho.org/english/gov/cd/ftcd_35.htm. Accessed 8 Nov 2017.

[3] Blanks J, Richards F, Beltran F, Collins R, Alvarez E, Zea Flores G (1998). The Onchocerciasis Elimination Program for the Americas: a history of partnership. Pan Am J Public Health..

[4] Sauerbrey M (2008). The Onchocerciasis Elimination Program for the Americas (OEPA). Ann Trop Med Parasitol..

[5] Duke BOL, Zea-Flores G, Castro J, Cupp EW, Muñoz B (1991). Comparison of the effects of a single dose and four six-monthly doses of ivermectin on adult *Onchocerca volvulus*. Am J Trop Med Hyg..

[6] Cupp EW, Duke BOL, Mackenzie CD, Guzmán JR, Vieira JC, Mendez-Galvan J (2004). The effects of long-term community-level treatment with ivermectin (Mectizan) on *Onchocerca volvulus* in Latin America. Am J Trop Med Hyg..

[7] Trapido H, D’Alessandro A, Little MD (1971). Onchocerciasis in Colombia. Historical background and ecologic observations. Am J Trop Med Hyg.

[8] Assis-Masri G, Little MD (1965). A case of ocular onchocerciasis in Colombia. Trans R Soc Trop Med Hyg..

[9] Little MD, D’Alessandro A (1970). Onchocerciasis in Colombia. Parasitologic findings in the first observed focus. Am J Trop Med Hyg..

[10] Lopez Villegas A, Allen JH, Little MD (1972). Onchocerciasis in Colombia: ocular findings in the first observed focus. Am J Trop Med Hyg..

[11] Barreto P, Trapido H, Lee VH (1970). Onchocerciasis in Colombia. Entomologic findings in the first observed focus. Am J Trop Med Hyg..

[12] Tidwell MA, Tidwell MA, Muñoz de Hoyos P, Corredor A (1980). *Simulium exiguum s.l.*, the vector of *Onchocerca volvulus* on the Rio Micay, Colombia. Am J Trop Med Hyg..

[13] Ewert A, Corredor A, Lightner L, D’Alessandro A (1979). Onchocerciasis focus in Colombia: follow-up after 12 years. Am J Trop Med Hyg..

[14] Palma GI, Travi BL, Satizabal JE, Martínez F, Smith DS (1995). Onchocerciasis in Colombia? A reassessment of the Lopez de Micay focus. Biomedica..

[15] Corredor A, Nicholls RS, Duque S, Muñoz P, Alvarez CA, Guderian RH (1998). Current status of onchocerciasis in Colombia. Am J Trop Med Hyg..

[16] Guderian JR, Anselmi M, Espinel M, Sandoval C, Cooper PJ, Rivadeneira G (1997). Onchocerciasis in Ecuador: prevalence of infection on the Ecuador-Colombia border in the Province of Esmeraldas. Mem Inst Oswaldo Cruz..

[17] World Health Organization (2001). Certification of elimination of Onchocerciasis. Criteria and procedures. Guidelines. WHO/CDS/CPE/CEE/2001.18b.

[18] Silva JC, Beltrán F, Semba R (1992). Ophtalmological assessment of onchocerciasis in the Americas. Proceedings of the workshop on ophtalmological assessment.

[19] Winthrop K, Proaño R, Oliva O, Arana B, Mendoza C, Dominguez A (2006). The reliability of anterior segment lesions as indicators of onchocercal eye disease in Guatemala. Am J Trop Med Hyg..

[20] Lopez HH, Corredor A, Nicholls RS, Alvarez CA, Palma GI (1997). Onchocerciasis: opthalmological assessment of the Colombian focus. Rev Soc Col Oft..

[21] Lipner EM, Dembele N, Souleymane S, Alley WS, Prevots DR, Toe L (2006). Field applicability of a rapid-format anti-Ov-16 antibody test for the assessment of onchocerciasis control measures in regions of endemicity. J Infect Dis..

[22] Lobos E, Weiss N, Karam M, Taylor HR, Ottesen EA, Nutman TB (1991). An immunogenic *Onchocerca volvulus* antigen: a specific and early marker of infection. Science..

[23] Lindblade KA, Arana B, Zea-Flores G, Rizzo N, Porter CH, Dominguez A (2007). Elimination of *Onchocerca volvulus* transmission in the Santa Rosa Focus of Guatemala. Am J Trop Med Hyg.

[24] Katholi CR, Toé L, Merriweather A, Unnasch TR (1975). Determining the prevalence of *Onchocerca volvulus* infection in vector populations by PCR screening of pools of black flies. J Infect Dis..

[25] World Health Organization (2016). Guidelines for stopping mass drug administration and verifying elimination of human onchocerciasis: criteria and procedures.

[26] World Health Organization (2013). Progress towards eliminating onchocerciasis in the WHO Region of the Americas: verification by WHO of elimination of transmission in Colombia. Wkly Epidemiol Rec..

[27] World Health Organization (2014). Elimination of onchocerciasis in the WHO Region of the Americas: Ecuador’s progress towards verification of elimination. Wkly Epidemiol Rec..

[28] Rodríguez-Pérez MA, Fernández-Santos NA, Orozco-Algarra ME, Rodríguez-Atanacio JA, Domínguez-Vázquez A, Rodríguez-Morales KB (2015). Elimination of onchocerciasis from Mexico. PLoS Negl Trop Dis..

[29] World Health Organization (2015). Progress toward eliminating onchocerciasis in the WHO Region of the Americas: verification of elimination of transmission in Mexico. Wkly Epidemiol Rec..

[30] Jr RF, Rizzo N, Diaz Espinoza CE, Monroy ZM, Crovella Valdez CG, de Cabrera RM (2015). One hundred years after its discovery in Guatemala by Rodolfo Robles, *Onchocerca volvulu*s transmission has been eliminated from the central endemic zone. Am J Trop Med Hyg..

[31] World Health Organization (2016). Progress towards eliminating onchocerciasis in the WHO Region of the Americas: verification of elimination of transmission in Guatemala. Wkly Epidemiol Rec..

[32] Katabarwa MN, Walsh F, Habomugisha P, Lakwo TL, Agunyo S, Oguttu DW (2012). Transmission of onchocerciasis in Wadelai focus of northwestern Uganda has been interrupted and the disease eliminated. J Parasitol Res..

[33] Katabarwa M, Lakwo T, Habomugisha P, Agunyo S, Byamukama E, Oguttu D (2014). Transmission of *Onchocerca volvulus* by *Simulium neavei* in Mount Elgon focus of Eastern Uganda has been interrupted. Am J Trop Med Hyg..

[34] Higazi TB, Zarroug IM, Mohamed HA, Elmubark WA, Deran TC, Aziz N (2013). Interruption of *Onchocerca volvulus* transmission in the Abu Hamed focus, Sudan. Am J Trop Med Hyg..

[35] Traore MO, Sarr MD, Badji A, Bissan Y, Diawara L, Doumbia K (2012). Proof-of principle of onchocerciasis elimination with ivermectin treatment in endemic foci in Africa: final results of a study in Mali and Senegal. PLoS Negl Trop Dis..

[36] Botto C, Basáñez MG, Escalona M, Vivas-Martínez S, Villamizar N, Noya-Alarcón O (2016). Evidence of suppression of onchocerciasis transmission in the Venezuelan Amazonian focus. Parasit Vectors..

